# Clinical outcomes of co-transfer of partially and fully compacted morulae versus a fully compacted morula alone on day 4

**DOI:** 10.3389/fendo.2026.1776207

**Published:** 2026-06-01

**Authors:** Lin-Lin Tao, Bo Zheng, Fang-Fang Dai, Ya-Song Geng, Guo-Zhen Li, Hao-Yang Dai, Zhi-Wei Yang, Shu-Song Wang, Jing Ma, Lingyin Kong

**Affiliations:** 1 Reproductive Center, Xingtai Meihe Reproductive and Genetic Hospital, Xingtai, Hebei, China; 2 Hebei Key Laboratory of Reproductive Medicine, Hebei Reproductive Health Hospital, Shijiazhuang, Hebei, China; 3 Basecare Medical Device Co., Ltd., Suzhou, China

**Keywords:** clinical outcomes, day 4 transfer, fresh cycles, fully compacted morula, partially compacted morula

## Abstract

**Objective:**

To evaluate the impact of day 4 double embryo transfer (DET) with a fully compacted morula (FCM) and a partially compacted morula (PCM) versus day 4 single embryo transfer (SET) with an FCM on clinical and neonatal outcomes in fresh cycles.

**Methods:**

This was a retrospective cohort study including 889 fresh day 4 embryo transfer cycles conducted between October 2018 and December 2024. Propensity score matching (PSM) was applied to control for potential confounders and compare the clinical outcomes between SET with FCM and DET with FCM and PCM.

**Results:**

After PSM, logistic regression analysis showed no significant differences in clinical pregnancy rate (CPR), miscarriage rate, live birth rate (LBR), cumulative live birth rate (CLBR), monozygotic twin rate, stillbirth rate, and cesarean section rate (CSR) (all *P* > 0.05). However, the DET with FCM and PCM group was associated with a significantly higher multiple pregnancy rate (MPR) (*P* < 0.001) and preterm delivery rate (PDR) (OR 4.02, 95% CI 1.75–9.22; *P* = 0.001) after matching. Among patients aged<35 years or undergoing IVF, the pregnancy and neonatal outcomes were consistent with the overall data presented above. Specifically, following logistic regression analysis, there were no significant differences in CPR, LBR, and CLBR between the two groups(all *P* > 0.05), but the MPR in the DET with FCM and PCM group was significantly higher(all *P*<0.001). Furthermore, after excluding early-stage blastocysts, the clinical and neonatal outcomes remained consistent with the main study findings.

**Conclusion:**

In fresh cycles, there were no significant differences in CPR, LBR and CLBR following day 4 DET with FCM and PCM compared with SET with FCM. However, the MPR was significantly higher in the DET group. SET with FCM may be a preferable strategy for balancing clinical and multiple pregnancy rates.

## Background

In recent years, assisted reproductive technologies (ART) have become widely used and continuous refined. These technologies encompass ovulation induction, embryo cryopreservation, embryo selection, and the ongoing optimization of embryo culture media. These advancements have made blastocyst transfer possible (1). Numerous studies have indicated that blastocysts have higher implantation and developmental potential compared with cleavage-stage embryos (2–4). An increasing number of fertility centers favor culturing embryos to the blastocyst stage to facilitate selective single blastocyst transfer (SBT). However, recent research has indicated that the compaction process during the morula stage involves multiple self-correcting mechanisms. These mechanisms are not only crucial for determining embryo quality (5), but also critical for subsequent embryonic development (6, 7). Several studies have shown that clinical pregnancy rate (CPR) and live birth rate (LBR) on day 4 of fresh IVF/ICSI cycles were similar to those on day 5 (8–11). Therefore, morula transfer could also serve as an alternative option for clinicians (9–11).

Multiple pregnancies have been reported to be associated with many obstetric and neonatal complications, such as hypertensive disorders of pregnancy, premature rupture of membranes, premature birth, postpartum hemorrhage and low birth weight (12, 13). The risk of preterm birth in twin pregnancies is six times that of singleton pregnancies, while the risk of low birth weight infants is up to ten times higher (14). The risk of perinatal mortality in twins pregnancies also increases fourfold (15). Therefore, the objective of ART is to achieve healthy singleton pregnancies, reduce the rate of multiple pregnancies, and maximize the cumulative live birth rate (CLBR) wherever possible (16).

The most effective measure for reducing multiple pregnancies is to lower the number of embryos transferred (17, 18), and elective single embryo transfer (eSET) is a strongly recommended strategy. SET during ART cycles significantly reduces multiple pregnancy rates (MPRs) and improves perinatal and neonatal outcomes, including caesarean section rates (CSRs), preterm delivery rates (PDRs), and low birth weight rates (19, 20). Embryo quality (based on morphological parameters) is a primary predictor of successful implantation and live birth. Apart from embryo quality, the number of embryos transferred is also a significant factor influencing pregnancy outcomes. Recent findings from a Cochrane systematic review suggested that women undergoing SET might have a lower LBR compared with those undergoing double embryo transfer (DET) (21). In practice, when only a single good-quality embryo (GQE) is available, clinicians may face a dilemma: should a poor-quality embryo (PQE) be transferred alongside the GQE to improve the CPR? Some studies have shown that the addition of a PQE to a GQE results in a significantly lower implantation rate (22) or an ongoing pregnancy rate (23). In contrast, the results of a systematic review and meta-analysis indicated that DET with PQE and GQE did not result in increased or decreased CPR or LBR compared with SET with GQE but led to a greater MPR (24). In addition, previous studies have shown that DET with PQE and GQE significantly increased the MPR, but did not increase the LBR (25, 26). However, Wang et al. (27) reported that adding PQE to GQE led to a significant increase in the LBR but also significantly increased the MPR. Therefore, there is currently no definitive conclusion regarding whether it is necessary to add a PQE to a GQE for transfer.

On day 4 after fertilization, the embryos are in the process of transitioning from the cleavage stage to the compacted morula stage. By this time, embryos have usually completed compaction and are characterized by tightly interconnected cells with ill-defined margins (28); in more rapidly developing embryos, a blastocyst cavity may even be observed. Embryonic compaction is essential for the formation of blastocyst trophoblast and inner cell mass (29). It has been reported that morula embryos with delayed and/or incomplete compaction have a reduced likelihood of developing into high-quality blastocysts (30). In addition, the partially compacted group had more pronounced developmental delay at the post-compaction stage, which may affect blastocyst formation, implantation and live birth. Partially compacted morulae (PCM) have a lower developmental potential compared with fully compacted morulae (FCM); however, the blastocysts formed from both groups demonstrate comparable clinical outcomes and normal ploidy rates (31). Cell exclusion, independent of the chromosome makeup of the excluded cells, may result from impaired embryonic development. The 2025 Istanbul Consensus (32) defines a FCM on day 4 as a high-quality embryo. Zhang et al. demonstrated that early blastocyst and FCM may result in higher pregnancy and live birth rates than PCM (19). There have also been some studies on the impact of day 4 embryo transfer on clinical outcomes. Although DET on day 4 had superior pregnancy outcomes, neonatal outcomes were poorer (10). Our previous research has shown that transferring two high-quality embryos on day 4 significantly increased the rate of multiple pregnancies. Therefore, we do not recommend transferring two high-quality embryos on day 4 (33). However, in clinical practice, clinicians sometimes aim to improve CPR and reduce MPR by employing DET with FCM and PCM. Compared with single FCM transfer, does DET with FCM and PCM affect clinical and neonatal outcomes? Is this approach beneficial in clinical practice? The purpose of this study was to evaluate the impact of day 4 DET with an FCM and a PCM versus day 4 SET of an FCM by using a propensity score matching (PSM) design to control for possible confounding factors.

## Materials and methods

### Study design and patients

This was a retrospective cohort study including 889 fresh day 4 embryo transfer cycles conducted at at the Reproductive Medicine Center of Xingtai Meihe Reproductive and Genetic Hospital between October 2018 and December 2024. These patients underwent embryo transfer on day 4 post-insemination, receiving either SET with FCM or DET with FCM and PCM. The exclusion criteria were as follows: (a) donor sperm or donor egg cycles; (b) cycles that did not use long or extra-long protocols; (c) rescue-ICSI cycles; (d) cycles with known uterine anomalies, including intrauterine adhesion, septal uterine cavity, endometriosis, adenomyosis and fibroids with a diameter larger than 4 cm; (e) cycles with uncontrolled endocrine or immune disorders; and (f) cycles with lost to follow-up. In this study, each couple was included only once. Patients were subsequently divided into two groups according to the quantity and quality of the embryos: the SET with FCM group and the DET with FCM and PCM group. To compare the clinical outcomes of the two groups, PSM was applied to control for potential confounders and selection biases. Furthermore, a subgroup analysis was conducted to examine the effect of co-transfer of PCM and FCM versus FCM alone on clinical and neonatal outcomes, with females stratified by age and insemination method. Due to the small sample size of cycles aged ≥35 years or those undergoing ICSI, this subgroup is included for exploratory purposes only. Finally, we performed sensitivity analyses to verify the robustness of the study results. The studies were approved by Xingtai Meihe Reproduction and Genetic Hospital (Protocol number 2023-ER-07). The studies were conducted in accordance with the local legislation and institutional requirements. Written informed consent for participation was not required from the participants or the participants’ legal guardians/next of kin in accordance with the national legislation and institutional requirements. This study was a retrospective study, and all patient information was anonymized and de-identified prior to analysis.

### IVF procedures and assessment

**Figure 1:**
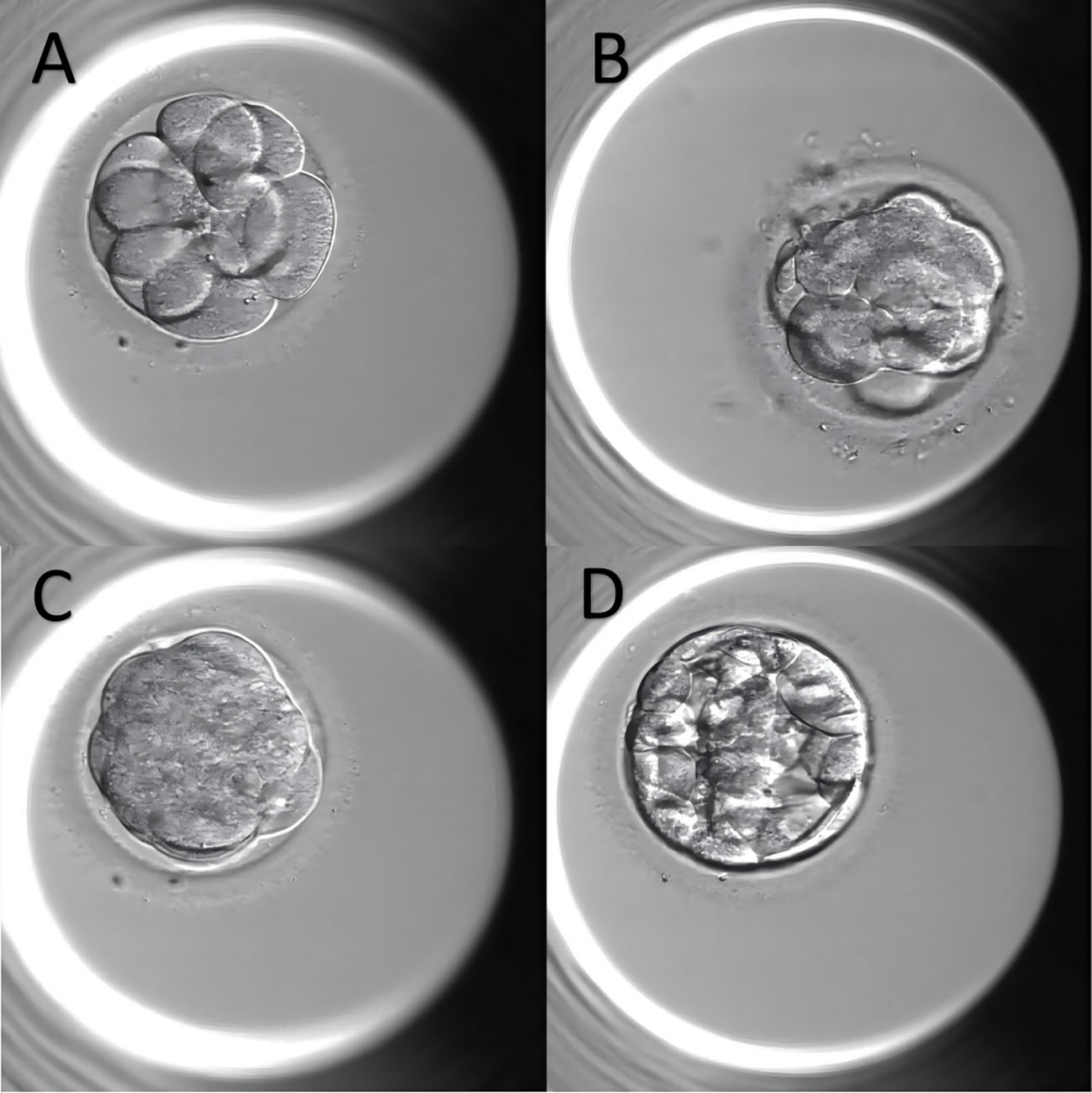
Embryonic development stages on day 4. **(A)** Cleavage-stage embryo; **(B)** Partially compacted morula; **(C)** Fully compacted morula; **(D)** Early-stage blastocyst.

Detailed information regarding ovulation induction and IVF/ICSI stimulation protocols has been described in our previous study (34). Conventional IVF or ICSI was performed depending on the semen parameters and previous fertilization history. The embryos were cultured at 37 °C under conditions of 6% CO2, 5% O2 and 89% N2 in G5 continuous medium (Vitrolife, Gothenburg, Sweden). Fertilization status was assessed approximately 16–18 hours after insemination. The 2011 ESHRE Istanbul Consensus (35) was used in our center to score embryos on day 4. On day 4, as the number of blastomeres in the embryo increases (**Figure 1A**), they begin to fuse together. The cell membrane boundaries between some cells become indistinct. However, as some cells have not fused, such an embryo is classified as PCM (**Figure 1B**). When all blastomeres have fused and the cell membrane boundaries are indistinct, the embryo resembles a single large cell. This is known as an FCM embryo (**Figure 1C**). As the embryo develops further, the number of cells increases significantly and spaces begin to form between them. This results in the formation of an early-stage (**Figure 1D**). Two technicians graded the embryos simultaneously. If there is a discrepancy in the assessment results, a third person will make the final decision. In this study, the term ‘FCM’ refers to embryos that have reached the fully compacted stage, including those in the early blastocyst phase. Serum β-hCG levels were measured 12–14 days after embryo transfer to determine biochemical pregnancy occurrence. Approximately four weeks post-transfer, transvaginal ultrasound was used to visualize the gestational sac and detect fetal cardiac activity. The number of gestational sacs was recorded, and the presence of a gestational sac was considered a clinical pregnancy. Luteal support continued until 12 weeks of gestation, and postpartum follow-up was conducted.

### Clinical outcomes

The primary outcome measure was clinical pregnancy rate. Secondary outcome variables included live birth rate, multiple pregnancy rate, and miscarriage rate, as well as neonatal outcomes. Neonatal outcomes included preterm birth rate, cesarean section rate, gestational age at delivery, and live birth weight. The clinical pregnancy rate was calculated by dividing the number of patients with at least one gestational sac detected by transvaginal ultrasound (performed 28 days after embryo transfer) by the total number of patients who underwent embryo transfer. A live birth was defined as a live baby delivered after 24 weeks of pregnancy. Multiple pregnancies were defined as the presence of multiple intrauterine fetuses. Miscarriage was defined as the loss of fetal cardiac activity within 28 weeks of confirming clinical pregnancy. Preterm birth was defined as a birth before 37 completed weeks of gestation. Cumulative live births were defined as the total number of live births achieved within a two-year period across all cycles following the current transfer, including the current fresh cycle and any subsequent frozen-thawed transfer cycles.

### Statistical analysis

Before conducting the data analysis, we reviewed the electronic or paper medical records to fill in all missing data. All data were statistically analyzed using SPSS 22.0 for Windows (IBM, Armonk, NY, USA). The data were examined for normality. Continuous variables that did not conform to a normal distribution were expressed as the median (25th, 75th percentile), M (Q1, Q3) and were compared using the Kruskal–Wallis test. Categorical variables were expressed as frequencies and proportions and were compared using the chi-square or Fisher’s exact test. *P*-values < 0.05 were considered statistically significant.

PSM was applied to control for potential confounders and selection biases (23). PSM was performed using logistic regression on potential outcome-related variables with the MatchIt package in R software (version 4.3.0). Patients were matched using a 1:1 nearest-neighbor matching algorithm without replacement, imposing a caliper of 0.1 on the logit of the propensity score. Standardized mean differences (SMDs) were used to assess covariate balance before and after matching, with SMD ≤ 0.1 indicating good balance. To further verify the results, multivariate logistic regression analyses were performed to adjust for the confounders mentioned above. Odds ratios (ORs) and 95% confidence intervals (95% CIs) were calculated from the model coefficients. The variables included the following variables: maternal age, maternal body mass index (BMI), basal follicle stimulating hormone (FSH), serum anti-Mullerian hormone (AMH) concentration, gonadotropins (Gn) dose used, estradiol (E_2_) level on the HCG day, luteinizing hormone (LH) level on the HCG day, progesterone (P) level on the HCG day, endometrial thickness, number of oocytes retrieved, pattern of infertility, infertility factors, ovarian stimulation protocol, pattern of insemination. Moreover, a subgroup analysis was performed to explore the impact of DET with FCM and PCM on clinical outcomes stratified by age and insemination method.

To address the low incidence of multiple pregnancies and avoid overfitting, we first performed LASSO regression to select candidate variables, forcing the coefficients of irrelevant factors to zero. We then applied Firth’s penalized logistic regression to the selected variables to obtain bias-corrected estimates and confidence intervals. For data sets with very few events, logistic regression analysis was not performed as the estimates were highly unstable. Only descriptive results are reported for these subgroups.

## Results

This study included a total of 889 cycles between October 2018 and December 2024.The SET with FCM group consisted of 645 cycles and the DET with FCM and PCM group included 244 cycles. After propensity score matching, 221 cycles in the SET with FCM group were matched with 221 cycles in the DET with FCM and PCM group.

**Figure 2:**
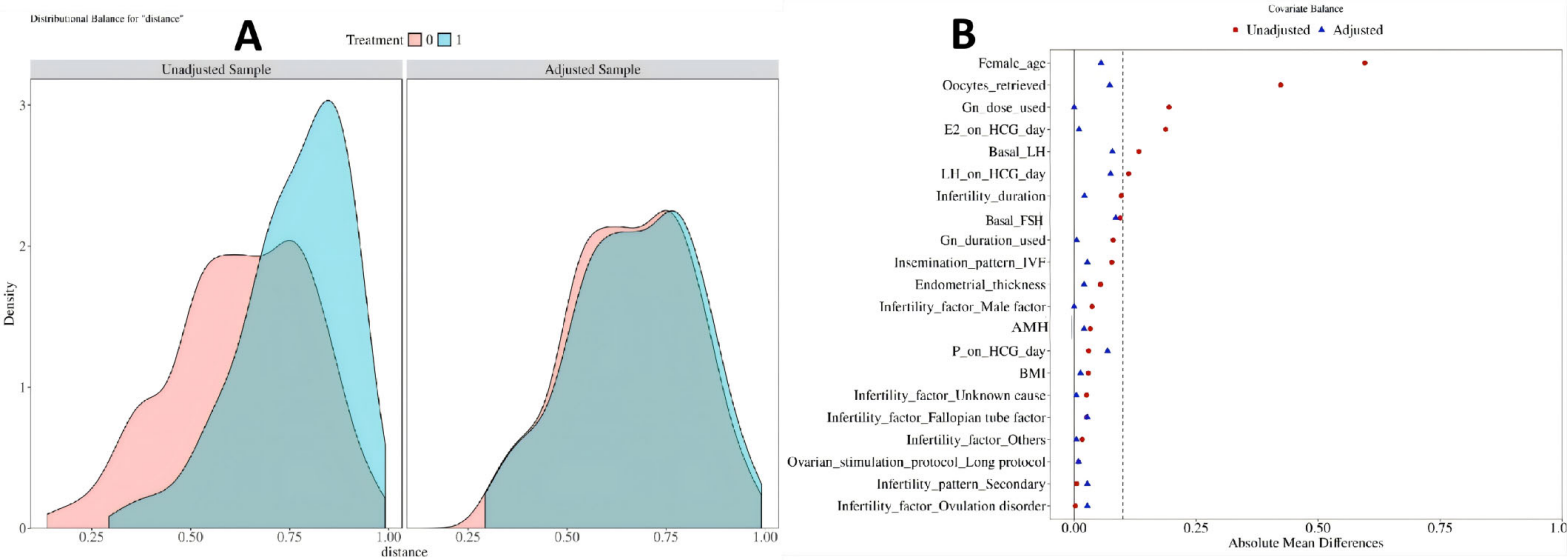
The balance of PSM. BMI, Body mass index; Gn, Gonadotropins; E_2_, Estradiol; LH, Luteinizing hormone; AMH, Antimullerian hormone; P, Progesterone; IVF, *In vitro* fertilization; PSM, Propensity score matching **(A)** Probability density analysis plots before and after PSM **(B)** Standardized mean difference of variables before and after PSM.

The patients’ overall baseline characteristics and IVF characteristics are presented in **Table 1** (left panel). As shown in **Table 1** (left panel), compared with the DET with FCM and PCM group before matching, patients who received SET with FCM were younger and had a lower Gn dose, higher E_2_ levels on the HCG day, a higher number of retrieved oocytes, and a higher percentage of cycles using IVF insemination. This is likely attributable to better ovarian reserve in these patients. **Table 1** (right panel) presents the comparison results after PSM. There were no statistically significant differences in baseline characteristics between the two groups (**Table 1**; **Figure 2**).

**Table 1:** Patient characteristics of the SET with FCM group and the DET with FCM and PCM group before and after PSM.

Variables	Before matching			After matching		
	FCM	FCM + PCM	*P*	FCM	FCM + PCM	*P*
Number of transfer cycles(n)	645	244		221	221	
Female age [years,M(Q1,Q3)]	30.00 (27.00, 33.00)	32.00 (29.00, 36.00)	<.001	32.00 (29.00, 34.00)	32.00 (29.00, 35.00)	0.708
Duration of infertility [years,M(Q1, Q3)]	3.00 (2.00, 5.00)	4.00 (2.00, 5.25)	0.154	4.00 (2.00, 6.00)	4.00 (2.00, 5.00)	0.647
Female BMI [kg/m2, M(Q1, Q3)]	24.30 (21.90, 26.90)	24.20 (21.50, 26.80)	0.783	24.40 (22.10, 26.70)	24.50 (21.60, 27.20)	0.691
Basal FSH[U/L,M(Q1,Q3)]	6.30 (5.30, 7.56)	6.30 (5.40, 7.93)	0.339	6.31 (5.16, 7.69)	6.29 (5.42, 7.86)	0.548
Basal LH[U/L,M(Q1,Q3)]	4.34 (3.02, 6.47)	4.18 (2.89, 5.97)	0.121	4.13 (2.94, 6.00)	4.27 (2.89, 6.04)	0.860
AMH[μg/L,M(Q1,Q3)]	3.67 (2.56, 5.26)	3.25 (2.29, 5.19)	0.065	3.75 (2.31, 5.17)	3.30 (2.30, 5.33)	0.397
Gn dose used[IU,M(Q1,Q3)]	2500.00 (2050.00, 3225.00)	2700.00 (2250.00, 3300.00)	0.010	2700.00 (2200.00, 3300.00)	2700.00 (2250.00, 3300.00)	0.871
Gn duration used[d,M(Q1,Q3)]	11.00 (10.00, 12.00)	11.00 (10.00, 12.00)	0.401	11.00 (11.00, 12.00)	11.00 (10.00, 12.00)	0.848
E2 on the HCG day [pg/ml, M (Q1, Q3)]	2788.00 (1960.00, 3811.00)	2528.00 (1741.00, 3384.75)	0.009	2529.00 (1805.00, 3530.00)	2606.00 (1763.00, 3530.00)	0.939
LH on the HCG day [U/L, M (Q1, Q3)]	0.98 (0.74, 1.34)	0.94 (0.71, 1.36)	0.348	0.92 (0.68, 1.26)	0.97 (0.71, 1.35)	0.360
P on the HCG day [ng/ml, M (Q1, Q3)]	0.72 (0.55, 0.90)	0.68 (0.51, 0.90)	0.544	0.72 (0.58, 0.91)	0.68 (0.50, 0.89)	0.394
Endometrial thickness[mm,M(Q1,Q3)]	11.50 (10.00, 13.00)	11.45 (10.00, 13.00)	0.528	11.50 (10.00, 13.00)	11.50 (10.00, 13.00)	0.968
Number of oocytes retrieved[n,M(Q1,Q3)]	14.00 (10.00, 18.00)	12.00 (9.00, 15.00)	<.001	13.00 (9.00, 16.00)	12.00 (9.00, 16.00)	0.418
Pattern of infertility(%)			0.883			0.555
Primary	37.83 (244/645)	37.30 (91/244)		35.75 (79/221)	38.46 (85/221)	
Secondary	62.17 (401/645)	62.70 (153/244)		64.25 (142/221)	61.54 (136/221)	
Proportion of infertility factors(%)			0.139			0.930
Unknown cause	5.43 (35/645)	2.87 (7/244)		3.62 (8/221)	3.17 (7/221)	
Male factor	6.98 (45/645)	10.66 (26/244)		10.86 (24/221)	10.86 (24/221)	
Ovulation disorder	14.57 (94/645)	14.34 (35/244)		11.76 (26/221)	14.48 (32/221)	
Fallopian tube factor	69.77 (450/645)	67.21 (164/244)		70.14 (155/221)	67.42 (149/221)	
Others	3.26(21/645)	4.92 (12/244)		3.62 (8/221)	4.07 (9/221)	
Ovarian stimulation protocol(%)			0.756			0.807
Extra-long protocol	16.74 (108/645)	17.62 (43/244)		18.10 (40/221)	19.00 (42/221)	
Long protocol	83.26(537/645)	82.38 (201/244)		81.90 (181/221)	81.00 (179/221)	
Pattern of insemination(%)			0.006			0.495
ICSI	14.42 (93/645)	22.13 (54/244)		23.98 (53/221)	21.27 (47/221)	
IVF	85.58 (552/645)	77.87 (190/244)		76.02 (168/221)	78.73 (174/221)	

Continuous variables were expressed as the median (25th, 75th percentile), M (Q1, Q3).

Categorical variables were expressed as frequencies (n) and proportions (%). P-values in bold depict statistical significance (*P* < 0.05) in comparison between SET with FCM and DET with FCM and PCM. SET, Single embryo transfer; DET, Double embryo transfer; PSM, Propensity score matching; FCM, Fully compacted morula; PCM, Partially compacted morula; IVF, *In vitro* fertilization; ICSI, Intracytoplasmic sperm injection; BMI, Body mass index; Gn, Gonadotropins; E_2_, Estradiol; LH, Luteinizing hormone; AMH, Antimullerian hormone; P, Progesterone.

**Table 2** shows the pregnancy and neonatal outcomes of two groups before and after PSM. After PSM, logistic regression analysis showed no significant differences in CPR, miscarriage rate, LBR, CLBR, monozygotic twin rate, stillbirth rate and cesarean section rate (CSR) (all *P* > 0.05). However, the DET with FCM and PCM group was associated with a significantly higher MPR (41.01% vs. 0.70%, *P* < 0.001) and PDR (OR 4.02, 95% CI 1.75–9.22; *P* = 0.001) after matching. In addition, compared with the PCM group, the DET with FCM and PCM group had a significantly lower gestational age (38 weeks vs. 39 weeks, *P* < 0.001) and a significantly lower live birth weight (2055 g vs. 3300 g, *P* < 0.001). However, there were no significant differences between the two groups in terms of gestational age or live birth weight at single births (*P*>0.05).

**Table 2:** Overall outcomes of the SET with FCM group and the DET with FCM and PCM group before and after PSM.

Variables	Before matching			After matching		
	FCM	FCM + PCM	*P*	FCM	FCM + PCM	*P*
Number of transfer cycles(n)	645	244		221	221	
Clinical pregnancy rate(%)	62.79 (405/645)	60.66 (148/244)	0.558	64.71 (143/221)	62.90 (139/221)	0.692
OR (95%CI)	Reference	0.94 (0.68-1.31)	0.723	Reference	0.88(0.59-1.32)	0.692
Miscarriage rate(%)	18.27 (74/405)	16.22 (24/148)	0.575	15.38 (22/143)	15.11 (21/139)	0.948
OR (95%CI)	Reference	0.84 (0.48-1.46)	0.541	Reference	1.06 (0.53-2.11)	0.867
Live birth rate(%)	51.01(329/645)	50.82 (124/244)	0.960	54.30 (120/221)	53.39 (118/221)	0.849
OR (95%CI)	Reference	1.03 (0.75-1.42)	0.836	Reference	0.94 (0.64-1.37)	0.735
Cumulative live birth rate(%)	72.40(467/645)	62.70(153/244)	0.005	68.78(152/221)	64.71(143/221)	0.364
OR (95%CI)	Reference	0.70(0.50-0.98)	0.036	Reference	0.82(0.54-1.22)	0.324
Multiple pregnancy rate(%)	0.49 (2/405)	41.89 (62/148)	< 0.001	0.70 (1/143)	41.01 (57/139)	< 0.001
OR (95%CI)	Reference	116.24 (37.98-576.40)	< 0.001	Reference	74.67 (19.24-673.81)	< 0.001
Monozygotic twin rate(%)	1.23 (5/405)	2.03 (3/148)	0.773	2.10 (3/143)	1.44 (2/139)	1.000
OR (95%CI)	Reference	1.18(0.21-6.79)	0.853	Reference	0.46 (0.04-5.12)	0.528
Premature delivery rate(%)	6.42 (26/405)	20.95 (31/148)	< 0.001	6.29 (9/143)	20.14 (28/139)	< 0.001
OR (95%CI)	Reference	3.80 (2.04-7.07)	< 0.001	Reference	4.02(1.75-9.22)	0.001
Stillbirth rate(%)	0.25(1/405)	0 (0/148)	0.545	0 (0/143)	0 (0/139)	/
Cesarean section rate(%)	49.88(202/405)	65.54 (97/148)	0.001	57.34 (82/143)	65.47 (91/139)	0.161
OR (95%CI)	Reference	1.68 (1.10-2.55)	0.016	Reference	1.36 (0.82-2.24)	0.235
Gestational week of delivery[weeks, M(Q1,Q3)]	39 (38, 39)	38.00 (36.75, 39)	< 0.001	39 (38, 40)	38 (37, 39)	0.001
Gestational age for a singleton[weeks, M(Q1,Q3)]	39(38, 39)	39(38, 40)	0.325	39(38, 39)	39(38, 39)	0.864
Live birth weight [g, M(Q1,Q3)]	3250 (3000, 3540)	2900 (2543, 3300)	< 0.001	3300(3000, 3500)	2055(2637.5, 3400)	<0.001
Live birth weight of a singleton[g, M(Q1,Q3)]	3300(3000, 3562.5)	3300(3050, 3500)	0.841	3300(3000, 3500)	3300(3000, 3500)	0.745

Continuous variables were expressed as the median (25th, 75th percentile), M (Q1, Q3).

Categorical variables were expressed as frequencies (n) and proportions (%). P-values in bold depict statistical significance (*P* < 0.05) in comparison between SET with FCM group and DET with FCM and PCM group. SET, Single embryo transfer; DET, Double embryo transfer; PSM, Propensity score matching; ORs, were adjusted for variables presented in the part of statistical analysis using multivariate logistic regression analyses before matching. ORs after matching were adjusted for propensity score.

FCM, Fully compacted morula; PCM, Partially compacted morula; OR, Odds ratios; CI, Confidence intervals.

**Figure 3:**
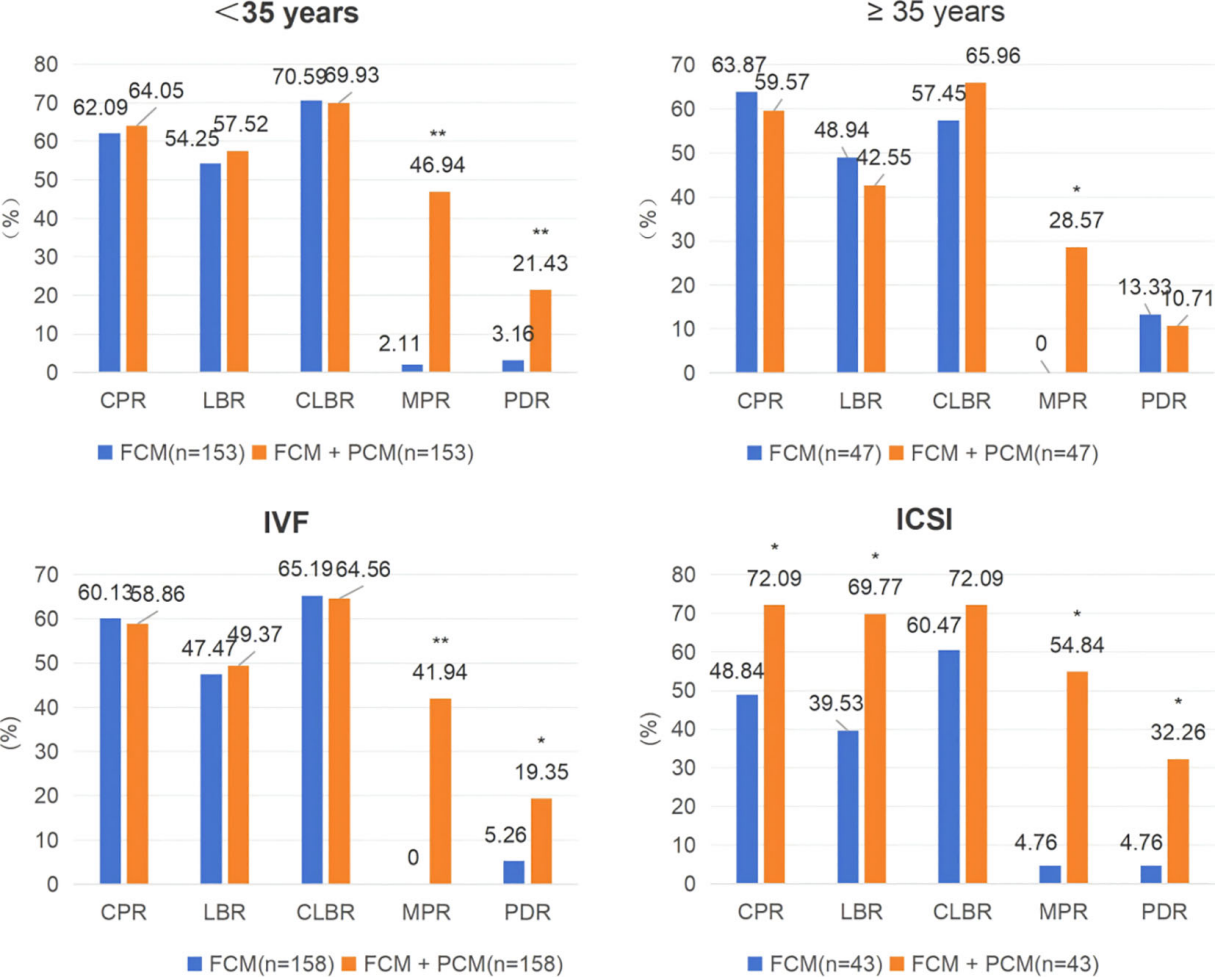
Outcomes of SET with FCM group and DET with FCM and PCM group by age and insemination method after PSM. * Indicates that following logistic regression analysis, compared with the FCM group, *P* < 0.05. ** Indicates that following logistic regression analysis, compared with the FCM group, *P* < 0.001. SET, Single embryo transfer; DET, Double embryo transfer; PSM, Propensity score matching; FCM, Fully compacted morula; PCM, Partially compacted morula; IVF, *In vitro* fertilization; ICSI, Intracytoplasmic sperm injection; CPR, Clinical pregnancy rate; LBR, Live birth rate; CLBR, Cumulative live birth rate; MPR, Multiple pregnancy rate; PDR, Premature delivery rate; PSM, Propensity score matching.

**Figure 4:**
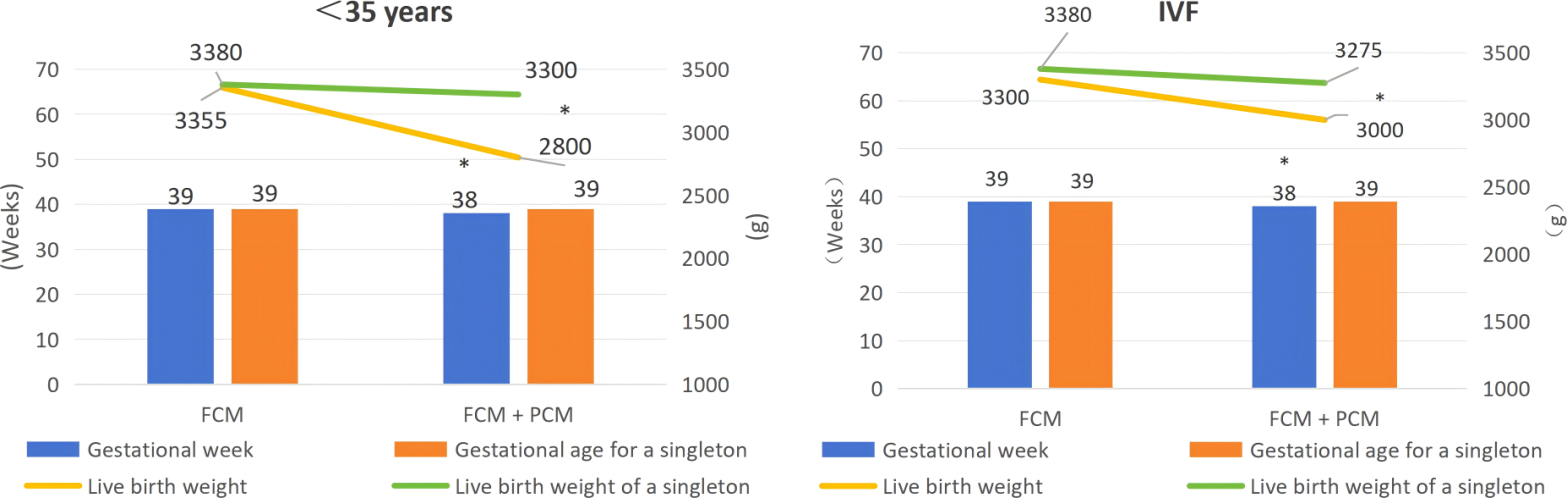
Gestational age and live birth weight of infants in patients <35 years or undergoing IVF after PSM. * Indicates that following logistic regression analysis, compared with the SET with FCM group, *P* < 0.05. FCM, Fully compacted morula; PCM, Partially compacted morula; IVF, *In vitro* fertilization; PSM, Propensity score matching.

To further investigate the impact of DET with FCM and PCM on pregnancy outcomes, we conducted subgroup analyses based on age and insemination method (**Figures 3**, **4**; **Supplementary Tables 1**, **2**). However, the sample sizes for patients aged ≥ 35 years or undergoing ICSI were small, and statistical power may have been insufficient. This part of the study was therefore exploratory in nature. Among patients aged<35, aged ≥ 35, or undergoing IVF, the pregnancy and neonatal outcomes were consistent with the overall data presented above. Specifically, following logistic regression analysis, there were no significant differences in CPR, LBR or CLBR between the two groups, but the MPR in the DET with FCM and PCM group was significantly higher (all *P*<0.05). In the ICSI cycles, the DET with FCM and PCM group showed significantly higher CPR (72.09% vs. 48.84%, *P* = 0.006) and LBR (69.77% vs. 39.53%, *P* = 0.002) than the FCM group; However, given the limited sample size at present, the statistical power may be insufficient. Validation with a larger sample size is required.

To eliminate the possibility of their influence on the results, we excluded early blastocysts from the analysis and conducted a sensitivity analysis. Following PSM, there were no significant differences in baseline characteristics between the two groups (**Supplementary Table 3**). The probability density analysis plots and standardized mean difference of variables before and after PSM are shown in **Supplementary Figure 1**. Consistent with the main findings, logistic regression analysis revealed no significant difference in CPR, LBR and CLBR between the two groups (*P*>0.05). However, the MPR was significantly higher in the DET with FCM and PCM group (39.29%) than in the FCM group (1.96%, *P*<0.001; **Supplementary Table 4**). This provides further support for the robustness of our findings.

## Discussion

This study indicates that there were no significant differences in CPR, LBR and CLBR following day 4 DET with FCM and PCM compared with SET with FCM. However, the MPR was significantly higher in the DET group. This was particularly evident in patients aged < 35 years and in those undergoing IVF. The sample sizes for patients aged ≥ 35 years and those undergoing ICSI were small, limiting these findings to exploratory analyses. Furthermore, after excluding early-stage blastocysts, the clinical and neonatal outcomes remained consistent with the main study findings. This provides further support for the robustness of our findings.

The SUN study showed that the day 4 DET group had better CPR and LBR than the SET group but had poorer neonatal outcomes. However, this study did not analyze embryo quality in subgroups (10). The 2025 Istanbul Consensus defined FCM on day 4 as a high-quality embryo (32). Furthermore, in line with the findings of Zhang et al. (19), our earlier study (34, 36) found that FCM transfer was associated with significantly higher CPR and LBR than PCM transfer. Additionally, neonatal outcomes following double FCM transfer were poorer than those following single FCM transfer. It remains unclear whether transferring DET with FCM and PCM results in better pregnancy outcomes than transferring a single FCM. Previous studies (22, 23) have found that DET with a PQE and a GQE significantly reduces the CPR. They proposed that during the biological process of implantation, the embryo interacts with the endometrium. Signaling crosstalk between embryos and the endometrium plays a crucial role in embryo implantation (37–39). Acting as a sensor for embryo quality, the endometrium recognizes signals emitted by embryos of varying quality. Decidualized endometrial stromal cells (ESCs), serving as biomarkers for arrested embryos, could impede implantation (40). Thus, the endometrium may be capable of distinguishing competitive embryos from those with developmental abnormalities, altering endometrial receptivity to protect the mother from the risks of abnormal pregnancy (41). The transfer of a PQE with a GQE might send aberrant or harmful signals to the endometrium, which further leads to adverse pregnancy outcomes. This study indicates that there were no significant differences in CPR, LBR and CLBR following day 4 DET with FCM and PCM compared with SET with FCM. However, the MPR was significantly higher in the DET group. This was particularly evident in patients aged < 35 years and in those undergoing IVF. Furthermore, after excluding early-stage blastocysts, the clinical and neonatal outcomes remained consistent with the main study findings. This provides further support for the robustness of our findings.

Consistent with the findings of this study, some studies have shown that DET with FCM and PCM did not significantly alter the CPR and LBR but did significantly improve the MPR compared with SET with FCM (24–26). Multiple pregnancies are associated with many obstetric and neonatal complications, including hypertensive disorders of pregnancy, preterm rupture of membranes, preterm birth, postpartum hemorrhage and low birth weight (12, 13). Our findings show that, compared with SET with FCM, DET with FCM and PCM significantly reduces live birth weight. However, there was no significant difference in singleton live birth weight between the two groups, suggesting that the lower live birth weight in the DET group may be attributable to its higher multiple pregnancy rate. Furthermore, DET did not increase the clinical pregnancy rate; therefore, adding a PCM to an FCM transfer may not achieve the objective of balancing clinical pregnancy and multiple pregnancies. A recent study (42) indicated that the addition of PQE in frozen-thawed cleavage-stage embryo transfer did not significantly improve LBR but increased MPR. For blastocyst-stage transfer, DET with FCM and PCM improved LBR but increased MPR, leading to adverse neonatal outcomes. In patients < 35 years old, SET with GQE yielded satisfactory LBR regardless of the embryonic development stage, while DET with GQE and PQE significantly increased the MPR. In patients aged ≥ 35 years old, selective SET appeared to be a more promising approach for reducing the risk of multiple live births and adverse neonatal outcomes. Differences in research findings may be attributed to a variety of factors, including age, embryonic stage, ovarian stimulation protocols, insemination methods, and culture media.

Therefore, to promote healthy singleton pregnancies and reduce the number of multiple pregnancies, SET with FCM on day 4 may be recommended. This is consistent with findings from studies on other embryonic stages. Multiple studies have indicated that for good-quality blastocysts, SBT is a superior choice regardless of maternal age (43). A recent study (44) suggested that for patients < 40 years, double SET at the blastocyst stage may result in higher LBR; for women aged ≥ 40 years, the LBR was comparable to that of DET. Furthermore, the overall rates of multiple pregnancy and complications were lower than those with DET. Double SET could be considered a first-line strategy among certain patient cohorts, including women of advanced maternal age, to improve reproductive outcomes and reduce the risk of morbidity following ART. However, the age cut-off for SET is a controversial issue. There are currently insufficient data to support the appropriate number of embryos to transfer in women of advanced maternal age (≥ 40 years). Some studies suggested that high-quality SET was preferred by women < 40 years (20, 45, 46). Given the higher incidence of oocyte aneuploidy and reduced uterine responsiveness in older women (47), the guidelines of the American Society for Reproductive Medicine (ASRM) (48) recommend multiple embryo transfer for older women. However, the exact age threshold for SET still needs to be confirmed by large-scale, multicenter studies. In this study, the number of patients aged ≥ 35 years was small, so an exploratory analysis was conducted. There were very few patients aged ≥ 40, so no specific analysis was carried out for this group. A dedicated study will be conducted once sufficient data have been collected.

The primary strength of this study lies in its pioneering investigation into should a co-transfer of PCM and FCM be performed on day 4 of the fresh cycle, providing evidence-based guidance for clinical transfer strategy selection. Additionally, this study employed PSM to control for potential confounding factors that could influence the results, given the significant differences in baseline characteristics between the overall groups. PSM provides a method for simulating random assignment as an alternative to randomized controlled trials (RCTs). Furthermore, comparisons were made not only across the overall group but also stratified by age and insemination method. Unfortunately, the study has several limitations that need to be taken into consideration. First, this was a retrospective cohort study. Although PSM was performed to evaluate the effects of co-transfer of PCM and FCM versus FCM alone on clinical outcomes, the sample size decreased after PSM, and the loss of unmatched cases might have unmeasured effects. Second, in clinical practice, clinicians may opt for DET when a patient’s prognosis is poor. Although this study employed PSM and logistic regression analysis to minimize differences between the two groups, it may still be difficult to fully correct for this selection bias. Third, the factors in this study include not only embryo quality but also the number of embryos transferred. This complexity makes it difficult to interpret the results. Fourth, the sample size for patients aged ≥ 35 years or undergoing ICSI is small, and statistical power may be insufficient. Consequently, The findings of this exploratory study should be interpreted with caution. Furthermore, as some positive events were relatively rare (such as multiple pregnancy), we employed Firth’s penalized logistic regression to reduce bias in the presence of rare events. However, penalized regression cannot fully compensate for the extreme sparsity of events. Consequently, the estimates may still be unstable, and these results should be interpreted with caution. Therefore, further large-scale randomized clinical trials and *in vitro* experimental studies are required to determine whether co-transfer of PCM and FCM on day 4 truly affects endometrial signaling and clinical outcomes.

In conclusion, this study indicated that there were no significant differences in CPR, LBR and CLBR following day 4 DET with FCM and PCM compared with SET with FCM. However, the MPR was significantly higher in the DET group. This was particularly evident in patients aged < 35 years and in those undergoing IVF. Furthermore, after excluding early-stage blastocysts, the clinical and neonatal outcomes remained consistent with the main study findings. Therefore, to promote healthy singleton pregnancies and reduce the number of multiple pregnancies, SET with FCM may be recommended. Further large-scale randomized clinical trials and *in vitro* experimental studies are required to determine whether co-transfer of PCM and FCM versus FCM alone on day 4 truly affects endometrial signaling and clinical outcomes.

## Data Availability

The original contributions presented in the study are included in the article/**Supplementary Material**. Further inquiries can be directed to the corresponding author.

## Supplementary material

Supplementary Table 1 Outcomes of the SET with FCM group and the DET with FCM and PCM group stratified by age before and after PSM Continuous variables were expressed as the median (25th, 75th percentile), M (Q1, Q3). Categorical variables were expressed as frequencies (n) and proportions (%).P-values in bold depict statistical significance (*P* < 0.05) in comparison between FCM and FCM + PCM.ORs were adjusted for variables presented in the part of statistical analysis using multivariate logistic regression analyses before matching. ORs after matching were adjusted for propensity score. SET, Single embryo transfer; DET, Double embryo transfer; PSM, Propensity score matching; FCM, Fully compacted morula; PCM, Partially compacted morula; OR, Odds ratios; CI, Confidence intervals.

Supplementary Table 2 Outcomes of the SET with FCM group and the DET with FCM and PCM group stratified by insemination method before and after PSM. Continuous variables were expressed as the median (25th, 75th percentile), M (Q1, Q3). Categorical variables were expressed as frequencies (n) and proportions (%).P-values in bold depict statistical significance (*P* < 0.05) in comparison between FCM and FCM + PCM.ORs were adjusted for variables presented in the part of statistical analysis using multivariate logistic regression analyses before matching. ORs after matching were adjusted for propensity score. SET, Single embryo transfer; DET, Double embryo transfer; PSM, Propensity score matching; FCM, Fully compacted morula; PCM, Partially compacted morula; OR, Odds ratios; CI, Confidence intervals.

Supplementary Table 3 Patient characteristics before and after PSM — excluding early-stage blastocyst.Continuous variables were expressed as the median (25th, 75th percentile), M (Q1, Q3). Categorical variables were expressed as frequencies (n) and proportions (%). P-values in bold depict statistical significance (*P* < 0.05) in comparison between two groups. PSM, Propensity score matching; FCM, Fully compacted morula; PCM, Partially compacted morula; IVF, *In vitro* fertilization; ICSI, Intracytoplasmic sperm injection; BMI, Body mass index; Gn, Gonadotropins; E_2_, Estradiol; LH, Luteinizing hormone; AMH, Antimullerian hormone; P, Progesterone.

Supplementary Table 4 Clinical outcomes of the two groups before and after PSM— excluding early-stage blastocysts. Continuous variables were expressed as the median (25th, 75th percentile), M (Q1, Q3). Categorical variables were expressed as frequencies (n) and proportions (%).P-values in bold depict statistical significance (*P* < 0.05) in comparison between two groups. PSM, Propensity score matching; ORs, were adjusted for variables presented in the part of statistical analysis using multivariate logistic regression analyses before matching. ORs after matching were adjusted for propensity score. PSM; Propensity score matching; FCM, Fully compacted morula; PCM, Partially compacted morula; OR, Odds ratios; CI, Confdence intervals.

Supplementary Figure 1 The balance of PSM BMI Body mass index, Gn Gonadotropins, E_2_ Estradiol, LH Luteinizing hormone, AMH Antimullerian hormone, P Progesterone, IVF *In vitro* fertilization, PSM Propensity score matching A:Probability density analysis plots before and after PSM B:Standardized mean difference of variables before and after PSM.
