# Correction: Clinical outcomes of co-transfer of partially and fully compacted morulae versus a fully compacted morula alone on day 4

**DOI:** 10.3389/fendo.2026.1900682

**Published:** 2026-06-19

**Authors:** Lin-Lin Tao, Bo Zheng, Fang-Fang Dai, Ya-Song Geng, Guo-Zhen Li, Hao-Yang Dai, Zhi-Wei Yang, Shu-Song Wang, Jing Ma, Lingyin Kong

**Affiliations:** 1 Reproductive Center, Xingtai Meihe Reproductive and Genetic Hospital, Xingtai, Hebei, China; 2 Hebei Key Laboratory of Reproductive Medicine, Hebei Reproductive Health Hospital, Shijiazhuang, Hebei, China; 3 Basecare Medical Device Co., Ltd., Suzhou, China

**Keywords:** clinical outcomes, day 4 transfer, fresh cycles, fully compacted morula, partially compacted morula

The title of this article was erroneously given as: “Clinical outcomes of co-transfer of partially and fully compacted morula versus fully compacted morula alone on day 4”. The correct title of the article is “Clinical outcomes of co-transfer of partially and fully compacted morulae versus a fully compacted morula alone on day 4”.

A correction has been made to the section **Materials and methods**, subsection *Study design and patients*. under The sample size of 889 was incorrectly stated as 898. This study is based on a sample size of 889; the tables and other data are correct.

The correct sentence is “This was a retrospective cohort study including 889 fresh day 4 embryo transfer cycles conducted at the Reproductive Medicine Center of Xingtai Meihe Reproductive and Genetic Hospital...”

A correction has been made to the section **Results**, paragraph 3, lines 5-6 “However, the DET with FCM and PCM group was associated with a significantly higher MPR (41.89% vs. 0.49%, *P* < 0.001) and…”

The correct statement is “However, the DET with FCM and PCM group was associated with a significantly higher MPR (41.01% vs. 0.70%, *P* < 0.001) and…”

A correction has been made to the section **Discussion**, paragraph 3, lines 8-12:

“Our findings showing that, compared with SET with FCM, DET with FCM and PCM significantly increases live birth weight. However, there was no significant difference in singleton live birth weight between the two groups, suggesting that the higher live birth weight in the DET group may be attributable to its higher multiple pregnancy rate.”

The correct statement is “Our findings show that, compared with SET with FCM, DET with FCM and PCM significantly reduces live birth weight. However, there was no significant difference in singleton live birth weight between the two groups, suggesting that the lower live birth weight in the DET group may be attributable to its higher multiple pregnancy rate.”

The original version of this article has been updated.

