# Supplementary material for: Clinical outcomes of co-transfer of partially and fully compacted morulae versus a fully compacted morula alone on day 4
Source: Front Endocrinol (Lausanne). 2026 Jun 1;17:1776207. doi: 10.3389/fendo.2026.1776207 (PMC13265299; doi:10.3389/fendo.2026.1776207)
Supplement: Supplementary Table 1 — Outcomes of the SET with FCM group and the DET with FCM and PCM group stratified by age before and after PSM Continuous variables were expressed as the median (25th, 75th percentile), M (Q1, Q3). Categorical variables were expressed as frequencies (n) and proportions (%).P-values in bold depict statistical significance (P < 0.05) in comparison between FCM and FCM + PCM.ORs were adjusted for variables presented in the part of statistical analysis using multivariate logistic regression analyses before matching. ORs after matching were adjusted for propensity score. SET, Single embryo transfer; DET, Double embryo transfer; PSM, Propensity score matching; FCM, Fully compacted morula; PCM, Partially compacted morula; OR, Odds ratios; CI, Confidence intervals. [file SupplementaryFile1.docx]

Supplementary Table 1 Outcomes of the SET with FCM group and the DET with FCM and PCM group stratified by age before and after PSM

| Variables | Before matching | | | After matching | | |
| --- | --- | --- | --- | --- | --- | --- |
|  | FCM | FCM + PCM | *P* | FCM | FCM + PCM | *P* |
| Age<35 |  |  |  |  |  |  |
| Number of transfer cycles(n) | 570 | 157 |  | 153 | 153 |  |
| Clinical pregnancy rate(%) | 62.98 (359/570) | 64.33 (101/157) | 0.756 | 62.09 (95/153) | 64.05 (98/153) | 0.722 |
| OR (95%CI) | Reference | 0.99 (0.67-1.45) | 0.962 | Reference | 1.16 (0.71-1.89) | 0.561 |
| Live birth rate(%) | 51.40 (293/570) | 57.32 (90/157) | 0.18 | 54.25 (83/153) | 57.52 (88/153) | 0.565 |
| OR (95%CI) | Reference | 1.17 (0.81-1.70) | 0.401 | Reference | 1.17 (0.73-1.88) | 0.511 |
| Cumulative live birth rate | 73.86(421/570) | 69.43(109/157) | 0.268 | 70.59(108/153) | 69.93(107/153) | 0.900 |
| OR (95%CI) | Reference | 0.73(0.49-1.10) | 0.133 | Reference | 0.97(0.58-1.62) | 0.919 |
| Multiple pregnancy rate(%) | 0.56 (2/359) | 46.53 (47/101) | < 0.001 | 2.11 (2/95) | 46.94 (46/98) | <0.001 |
| Premature delivery rate(%) | 6.13 (22/359) | 21.78 (22/101) | < 0.001 | 3.16 (3/95) | 21.43 (21/98) | <0.001 |
| OR (95%CI) | Reference | 4.44 (2.22-8.88) | < 0.001 | Reference | 11.42 (2.94-44.31) | <0.001 |
| Cesarean section rate(%) | 50.42 (181/359) | 68.32 (69/101) | 0.001 | 51.58 (49/95) | 69.39 (68/98) | 0.011 |
| OR (95%CI) | Reference | 1.85 (1.13-3.05) | 0.015 | Reference | 1.94 (1.01-3.75) | 0.050 |
| Gestational week[weeks, M(Q1,Q3)] | 39.00 (38.00, 39.00) | 38.00 (37.00, 39.00) | 0.002 | 39(38, 40) | 38(37, 39) | <0.001 |
| Gestational age for a singleton[weeks, M(Q1,Q3)] | 39(38, 39) | 39(38, 39.25) | 0.503 | 39(38, 38) | 39 (37, 39.5) | 0.913 |
| Live birth weight [g, M(Q1,Q3)] | 3250 (3000, 3508) | 2800 (2510, 3238) | <.001 | 3355 (3000, 3555) | 2800 (2520, 3225) | <0.001 |
| Live birth weight of a singleton, [g, M(Q1,Q3)] | 3300(3000, 3562.5) | 3300(3037.5, 3500) | 0.881 | 3380(3000, 3025) | 3300(3565, 3500) | 0.477 |
| Age ≥35 |  |  |  |  |  |  |
| Number of transfer cycles(n) | 75 | 87 |  | 47 | 47 |  |
| Clinical pregnancy rate(%) | 61.33 (46/75) | 54.02 (47/87) | 0.348 | 63.83 (30/47) | 59.57 (28/47) | 0.671 |
| OR (95%CI) | Reference | 0.92(0.43-1.97) | 0.830 | Reference | 0.77(0.30-1.97) | 0.589 |
| Live birth rate(%) | 48.00 (36/75) | 39.08 (34/87) | 0.253 | 48.94 (23/47) | 42.55 (20/47) | 0.535 |
| OR (95%CI) | Reference | 0.93 (0.43-2.00) | 0.857 | Reference | 0.72 (0.29-1.77) | 0.469 |
| Cumulative live birth rate(%) | 61.33(46/75) | 50.57(44/87) | 0.169 | 57.45(27/47) | 65.96(31/47) | 0.396 |
| OR (95%CI) | Reference | 1.71(0.50-5.89) | 0.396 | Reference | 1.54(1.61-3.89) | 0.362 |
| Multiple pregnancy rate(%) | 0.00 (0/46) | 31.91 (15/47) | < 0.001 | 0.00 (0/30) | 28.57 (8/28) | 0.004 |
| Premature delivery rate(%) | 8.70 (4/46) | 19.15 (9/47) | 0.146 | 13.33 (4/30) | 10.71 (3/28) | 0.705 |
| OR (95%CI) | Reference | 1.98 (0.36-10.96) | 0.432 | Reference | 0.13(0.00-19.25) | 0.423 |
| Cesarean section rate(%) | 45.65 (21/46) | 59.57 (28/47) | 0.179 | 46.67 (14/30) | 57.14 (16/28) | 0.614 |
| OR (95%CI) | Reference | 1.75 (0.65-4.76) | 0.271 | Reference | 1.88 (0.52-6.86) | 0.339 |
| Gestational week of delivery[weeks, M(Q1,Q3)] | 38 (37, 39) | 38 (36.25, 39) | 0.336 | 38(37, 39) | 38.5(37, 39) | 0.813 |
| Gestational age for a singleton[weeks, M(Q1,Q3)] | 38 (37, 39) | 39 (37.25, 39) | 0.658 | 38 (37, 39) | 39 (37, 39) | 0.854 |
| Live birth weight [g, M(Q1,Q3)] | 3315 (2935, 3563) | 3000 (2660, 3440) | 0.064 | 3400 (3000, 3650) | 3300 (3000, 3500) | 0.646 |
| Live birth weight of a singleton [g, M(Q1,Q3)] | 3315 (2935, 3562.5) | 3250(2900, 3400) | 0.736 | 3400(3000, 3650) | 3300(3000, 3500) | 0.646 |

Continuous variables were expressed as the median (25th, 75th percentile), M (Q1, Q3).

Categorical variables were expressed as frequencies (n) and proportions (%).P-values in bold depict statistical significance (*P* < 0.05) in comparison between FCM and FCM + PCM.ORs were adjusted for variables presented in the part of statistical analysis using multivariate logistic regression analyses before

matching. ORs after matching were adjusted for propensity score

SET Single embryo transfer, DET Double embryo transfer, PSM Propensity score matching, FCM Fully compacted morula , PCM Partially compacted morula , OR Odds ratios, CI Confdence intervals

Supplementary Table 2 Outcomes of the SET with FCM group and the DET with FCM and PCM group stratified by insemination method before and after PSM

| Variables | Before matching | | | After matching | | |
| --- | --- | --- | --- | --- | --- | --- |
|  | FCM | FCM + PCM | *P* | FCM | FCM + PCM | *P* |
| IVF |  |  |  |  |  |  |
| Number of transfer cycles(n) | 552 | 190 |  | 158 | 158 |  |
| Clinical pregnancy rate(%) | 63.22 (349/552) | 57.89 (110/190) | 0.192 | 95 (60.13) | 93 (58.86) | 0.819 |
| OR (95%CI) | Reference | 0.79 (0.55-1.13) | 0.190 | Reference | 0.91 (0.57-1.47) | 0.707 |
| Live birth rate(%) | 51.45 (284/552) | 46.84 (89/190) | 0.273 | 75 (47.47) | 78 (49.37) | 0.736 |
| OR (95%CI) | Reference | 0.85 (0.59-1.21) | 0.357 | Reference | 1.07 (0.67-1.71) | 0.771 |
| Cumulative live birth rate(%) | 72.46(400/552) | 61.58(117/190) | 0.005 | 65.19(103/158) | 64.56(102/158) | 0.906 |
| OR (95%CI) | Reference | 0.66(0.45-0.95) | 0.028 | Reference | 0.96(0.59-1.57) | 0.885 |
| Multiple pregnancy rate(%) | 0.29 (1/349) | 39.09 (43/110) | < 0.001 | 0 (0.00) | 39 (41.94) | < 0.001 |
| Premature delivery rate(%) | 6.59 (23/349) | 19.09 (21/110) | < 0.001 | 5 (5.26) | 18 (19.35) | 0.003 |
| OR (95%CI) | Reference | 3.45 (1.71-6.97) | < 0.001 | Reference | 5.07 (1.69-15.25) | 0.004 |
| Cesarean section rate(%) | 50.72 (177/349) | 62.73 (69/110) | 0.028 | 51 (53.68) | 61 (65.59) | 0.096 |
| OR (95%CI) | Reference | 1.49 (0.93-2.39) | 0.101 | Reference | 1.78 (0.94-3.39) | 0.078 |
| Gestational week of delivery [weeks, M(Q1,Q3)] | 39.00 (38.00, 39.00) | 38.00 (37.00, 39.00) | 0.004 | 39 (38, 39.5) | 38(37, 39) | 0.005 |
| Gestational age for a singleton, M(Q1,Q3)] | 39.00 (38.00, 39.00) | 39.00 (38.00, 39.00) | 0.802 | 39 (38, 39.5) | 39 (38, 39) | 0.620 |
| Live birth weight [g, M(Q1,Q3)] | 3250 (3000, 3510) | 2900 (2520, 3300) | < 0.001 | 3300 (3000, 3695) | 3000 (2600, 3400) | < 0.001 |
| Live birth weight of a singleton, [weeks,M(Q1,Q3)] | 3300(3000, 3565) | 3300(3000, 3500) | 0.796 | 3300 (3000, 3695) | 3275(3000, 3500) | 0.562 |
| ICSI |  |  |  |  |  |  |
| Number of transfer cycles(n) | 93 | 54 |  | 43 | 43 |  |
| Clinical pregnancy rate(%) | 60.22 (56/93) | 70.37 (38/54) | 0.216 | 48.84 (21/43) | 72.09 (31/43) | 0.027 |
| OR (95%CI) | Reference | 2.25 (0.89-5.64) | 0.085 | Reference | 7.90(1.79-34.87) | 0.006 |
| Live birth rate(%) | 48.39 (45/93) | 64.81 (35/54) | 0.054 | 39.53 (17/43) | 69.77 (30/43) | 0.005 |
| OR (95%CI) | Reference | 2.93 (1.25-6.89) | 0.013 | Reference | 7.48 (2.10-26.67) | 0.002 |
| Cumulative live birth rate(%) | 72.04(67/93) | 66.67(36/54) | 0.493 | 60.47(26/43) | 72.09(31/43) | 0.254 |
| OR (95%CI) | Reference | 1.11(0.47-2.62) | 0.817 | Reference | 2.38(0.79-7.14) | 0.123 |
| Multiple pregnancy rate(%) | 1.79 (1/56) | 50.00 (19/38) | <0.001 | 4.76 (1/21) | 54.84 (17/31) | <0.001 |
| Premature delivery rate(%) | 5.36 (3/56) | 26.32 (10/38) | 0.004 | 4.76 (1/21) | 32.26 (10/31) | 0.042 |
| OR (95%CI) | Reference | 29.65 (2.84-309.62) | 0.005 | Reference | / | / |
| Cesarean section rate(%) | 44.64 (25/56) | 73.68 (28/38) | 0.005 | 10 (47.62) | 23 (74.19) | 0.051 |
| OR (95%CI) | Reference | 3.51(1.10-11.23) | 0.035 | Reference | 3.29 (0.58-18.73) | 0.179 |
| Gestational week of delivery [weeks, M(Q1,Q3)] | 39(38, 39) | 38(36, 39) | 0.033 | 39(38, 39) | 37.5(36, 39) | 0.058 |
| Gestational age for a singleton[weeks, M(Q1,Q3)] | 39(38, 39) | 39(39, 39) | 0.176 | 39(38, 39) | 39(39, 39) | 0.368 |
| Live birth weight [g, M(Q1,Q3)] | 3350 (3000, 3550) | 2800 (2600, 3250) | <.001 | 3400 (3200, 3600) | 2800 (2580, 3200) | <.001 |
| Live birth weight of a singleton[g, M(Q1,Q3)] | 3350 (3000, 3550) | 3200(3050, 3500) | 0.547 | 3400 (3200, 3600) | 3200(3050, 3500) | 0.449 |

Continuous variables were expressed as the median (25th, 75th percentile), M (Q1, Q3).

Categorical variables were expressed as frequencies (n) and proportions (%).P-values in bold depict statistical significance (*P* < 0.05) in comparison between FCM and FCM + PCM.ORs were adjusted for variables presented in the part of statistical analysis using multivariate logistic regression analyses before

matching. ORs after matching were adjusted for propensity score

SET Single embryo transfer, DET Double embryo transfer, PSM Propensity score matching, FCM Fully compacted morula , PCM Partially compacted morula , OR Odds ratios, CI Confdence intervals

Supplementary Table 3 Patient characteristics before and after PSM — excluding early-stage blastocyst

| Variables | Before matching | | | After matching | | |
| --- | --- | --- | --- | --- | --- | --- |
|  | FCM | FCM + PCM | *P* | FCM | FCM + PCM | *P* |
| Number of transfer cycles(n) | 120 | 134 |  | 91 | 91 |  |
| Female age [years,M(Q1,Q3)] | 31.00 (28.00, 33.00) | 31.00 (28.00, 34.00) | 0.463 | 31.00 (27.00, 33.00) | 30.00 (28.00, 34.00) | 0.839 |
| Duration of infertility [years,M(Q1, Q3)] | 3.00 (2.00, 5.00) | 3.00 (2.00, 5.00) | 0.875 | 3.00 (2.00, 5.00) | 4.00 (2.00, 5.00) | 0.238 |
| Female BMI [kg/m2, M(Q1, Q3)] | 23.95 (22.00, 25.80) | 24.20 (21.55, 26.67) | 0.386 | 23.90 (22.05, 25.85) | 24.20 (21.60, 26.65) | 0.6 |
| Basal FSH[U/L,M(Q1,Q3)] | 6.24 (5.30, 7.56) | 6.47 (5.54, 8.04) | 0.182 | 6.46 (5.43, 7.70) | 6.57 (5.54, 8.00) | 0.49 |
| Basal LH[U/L,M(Q1,Q3)] | 4.16 (3.05, 6.27) | 4.33 (3.02, 6.39) | 0.945 | 4.15 (3.05, 6.15) | 4.33 (3.06, 6.24) | 0.792 |
| AMH[μg/L,M(Q1,Q3)] | 3.66 (2.72, 5.05) | 3.60 (2.50, 5.91) | 0.799 | 3.66 (2.63, 5.01) | 3.16 (2.25, 5.07) | 0.332 |
| Gn dose used[IU,M(Q1,Q3)] | 2587.50 (2025.00, 3168.75) | 2650.00 (2131.25, 3150.00) | 0.534 | 2675.00 (2025.00, 3150.00) | 2625.00 (2212.50, 3087.50) | 0.937 |
| Gn duration used[d,M(Q1,Q3)] | 11.00 (10.00, 12.25) | 11.50 (10.25, 12.00) | 0.556 | 11.00 (10.00, 13.00) | 12.00 (10.50, 12.00) | 0.93 |
| E2 on the HCG day [pg/ml, M (Q1, Q3)] | 2990.50 (2003.75, 3899.50) | 2643.50 (1977.00, 3632.25) | 0.101 | 2879.00 (1963.50, 3813.50) | 2744.00 (2000.00, 3843.50) | 0.945 |
| LH on the HCG day [U/L, M (Q1, Q3)] | 1.05 (0.79, 1.30) | 0.96 (0.72, 1.36) | 0.219 | 1.05 (0.78, 1.29) | 0.98 (0.73, 1.33) | 0.453 |
| P on the HCG day [ng/ml, M (Q1, Q3)] | 0.73 (0.56, 0.89) | 0.67 (0.51, 0.92) | 0.754 | 0.74 (0.56, 0.91) | 0.73 (0.56, 0.90) | 0.835 |
| Endometrial thickness[mm,M(Q1,Q3)] | 11.90 (10.00, 13.00) | 11.40 (10.00, 13.00) | 0.285 | 12.00 (10.00, 13.00) | 11.30 (10.00, 13.00) | 0.618 |
| Number of oocytes retrieved [n,M(Q1,Q3)] | 13.50 (10.00, 17.00) | 12.00 (9.00, 16.00) | 0.129 | 13.00 (10.00, 17.00) | 12.00 (9.00, 16.00) | 0.933 |
| Pattern of infertility(%) |  |  | 0.091 |  |  | 0.758 |
| Primary | 30.83 (37/120) | 41.04 (55/134) |  | 37.36 (34/91) | 35.16 (32/91) |  |
| Secondary | 69.17 (83/120) | 58.96 (79/134) |  | 62.64 (57/91) | 64.84 (59/91) |  |
| Proportion of infertility factors(%) |  |  | 0.169 |  |  | 0.981 |
| Unknown cause | 2.50 (3/120) | 2.24 (3/134) |  | 3.30(3/91) | 3.30 (3/91) |  |
| Male factor | 9.17 (11/120) | 14.18 (19/134) |  | 10.99 (10/91) | 13.19 (12/91) |  |
| Ovulation disorder | 10.83 (13/120) | 17.16 (23/134) |  | 12.09 (11/91) | 12.09 (11/91) |  |
| Fallopian tube factor | 70.83 (85/120) | 64.18 (86/134) |  | 71.43 (65/91) | 68.13 (62/91) |  |
| Others | 6.67 (8/120) | 2.24 (3/134) |  | 2.20 (2/91) | 3.30 (3/91) |  |
| Ovarian stimulation protocol(%) |  |  | 0.916 |  |  | 0.83 |
| Extra-long protocol | 16.67 (20/120) | 17.16 (23/134) |  | 14.29 (13/91) | 13.19 (12/91) |  |
| Long protocol | 83.33 (100/120) | 82.84 (111/134) |  | 85.71 (78/91) | 86.81 (79/91) |  |
| Pattern of insemination(%) |  |  | 0.156 |  |  | 0.864 |
| ICSI | 20.00 (24/120) | 27.61 (37/134) |  | 24.18 (22/91) | 25.27 (23/91) |  |
| IVF | 80.00 (96/120) | 72.39 (97/134) |  | 75.82 (69/91) | 74.73 (68/91) |  |

Continuous variables were expressed as the median (25th, 75th percentile), M (Q1, Q3).

Categorical variables were expressed as frequencies (n) and proportions (%). P-values in bold depict statistical significance (*P* < 0.05) in comparison between two groups. PSM Propensity score matching, FCM Fully compacted morula, PCM Partially compacted morula, IVF In vitro fertilization, ICSI Intracytoplasmic sperm injection, BMI Body mass index, Gn Gonadotropins, E_2_ Estradiol, LH Luteinizing hormone, AMH Antimullerian hormone, P Progesterone


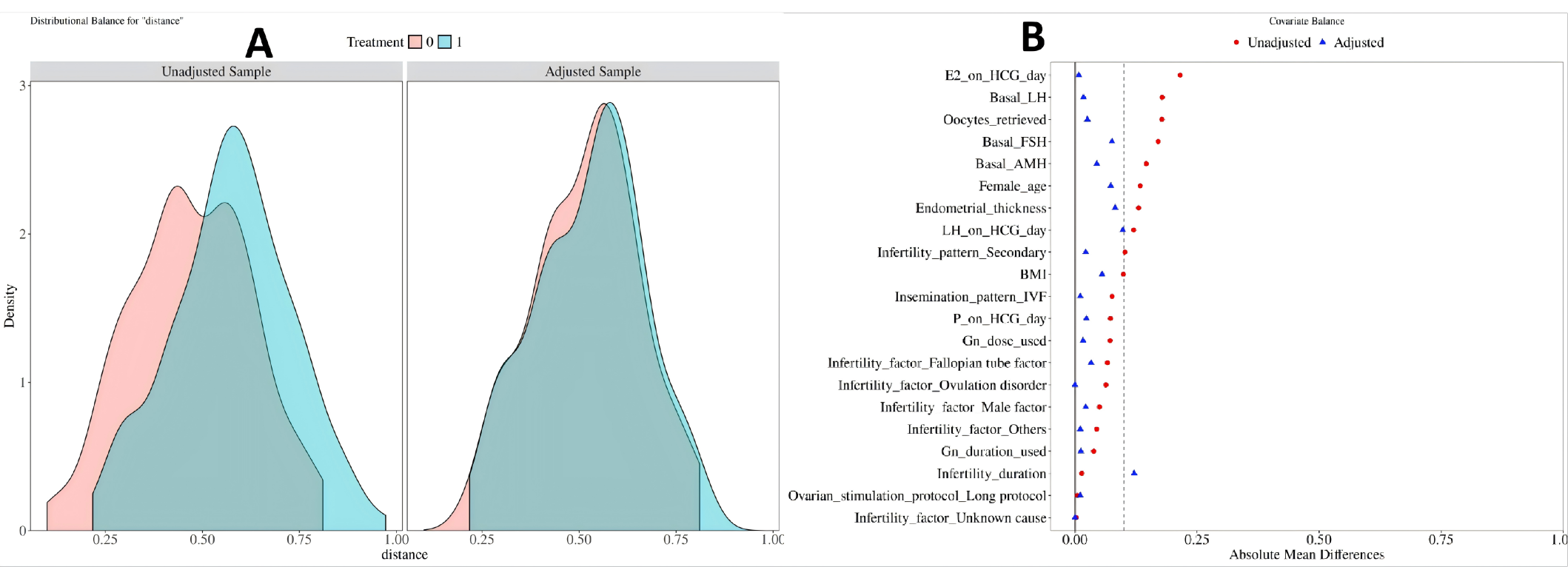


Supplementary Fig 1. The balance of PSM

BMI Body mass index, Gn Gonadotropins, E_2_ Estradiol, LH Luteinizing hormone, AMH Antimullerian hormone, P Progesterone, IVF In vitro fertilization, PSM Propensity score matching

A：Probability density analysis plots before and after PSM

B：Standardized mean difference of variables before and after PSM.

Supplementary Table 4 Clinical outcomes of the two groups before and after PSM— excluding early-stage blastocysts

| Variables | Before matching | | | After matching | | |
| --- | --- | --- | --- | --- | --- | --- |
|  | FCM | FCM + PCM | *P* | FCM | FCM + PCM | *P* |
| Number of transfer cycles(n) | 120 | 134 |  | 91 | 91 |  |
| Clinical pregnancy rate(%) | 59.17(71/120) | 63.43 (85/134) | 0.486 | 56.04 (51/91) | 61.54 (56/91) | 0.451 |
| OR (95%CI) | Reference | 1.15(0.66-2.00) | 0.619 | Reference | 1.26(0.68-2.35) | 0.496 |
| Miscarriage rate(%) | 16.90 (12/71) | 15.29 (13/85) | 0.785 | 17.65 (9/51) | 14.29 (8/56) | 0.635 |
| OR (95%CI) | Reference | 0.95(0.36-2.53) | 0.916 | Reference | 0.75(0.22-2.59) | 0.649 |
| Live birth rate(%) | 48.33 (58/120) | 53.73 (72/134) | 0.390 | 45.05 (41/91) | 52.75 (48/91) | 0.299 |
| OR (95%CI) | Reference | 1.23(0.72-2.11) | 0.456 | Reference | 1.45(0.78-2.69) | 0.241 |
| Cumulative live birth rate(%) | 66.67 (80/120) | 62.69 (84/134) | 0.508 | 61.54 (56/91) | 60.44 (55/91) | 0.879 |
| OR (95%CI) | Reference | 0.78(0.44-1.38) | 0.387 | Reference | 0.96(0.51-1.82) | 0.898 |
| Multiple pregnancy rate(%) | 1.41 (1/71) | 44.71 (38/85) | <.001 | 1.96 (1/51) | 39.29 (22/56) | <.001 |
| OR (95%CI) | Reference | 48.68 (11.30-459.70) | <.001 | Reference | 40.21 (7.67-512.24) | <.001 |
| Monozygotic twin rate(%) | 2.82 (2/71) | 2.35 (2/85) | 1.000 | 3.92 (2/51) | 1.79 (1/56) | 0.934 |
| OR (95%CI) | Reference | 0.77(0.04-14.00) | 0.861 | Reference | 1.68(0.00-20731.77) | 0.914 |
| Premature delivery rate(%) | 7.04 (5/71) | 23.53 (20/85) | 0.005 | 5.88 (3/51) | 23.21 (13/56) | 0.012 |
| OR (95%CI) | Reference | 4.21(1.28-13.87) | 0.18 | Reference | 6.53(1.35-31.56) | 0.02 |
| Stillbirth rate(%) | 1.41 (1/71) | 0 (0/134) | 0.545 | 0 (0/91) | 0 (0/91) | / |
| Cesarean section rate(%) | 41 (57.75) | 62.35 (53/85) | 0.558 | 52.94 (27/51) | 60.71 (34/56) | 0.417 |
| OR (95%CI) | Reference | 1.23(0.59-2.55) | 0.581 | Reference | 1.57(0.66-3.72) | 0.308 |
| Gestational week of delivery[weeks, M(Q1,Q3)] | 39 (38, 39) | 38 (36, 39) | 0.080 | 39 (38, 39) | 38 (36, 39) | 0.024 |
| Gestational age for a singleton[weeks, M(Q1,Q3)] | 39(38, 39) | 39(38, 40) | 0.109 | 39(38, 39.25) | 39(38, 39.75) | 0.995 |
| Live birth weight [g, M(Q1,Q3)] | 3150 (3000, 3400) | 2800 (2500, 3250) | < 0.001 | 3300(2900, 3400) | 2800(2500 3300) | 0.004 |
| Livge birth weight of a singleton[g, M(Q1,Q3)] | 3200(3000, 3400) | 3250(3012.5, 3500) | 0.368 | 3300(3000, 3400) | 3275(3052.5, 3500) | 0.524 |

Continuous variables were expressed as the median (25th, 75th percentile), M (Q1, Q3).

Categorical variables were expressed as frequencies (n) and proportions (%).P-values in bold depict statistical significance (*P* < 0.05) in comparison between two groups. PSM Propensity score matching, ORs were adjusted for variables presented in the part of statistical analysis using multivariate logistic regression analyses before matching. ORs after matching were adjusted for propensity score

PSM Propensity score matching, FCM Fully compacted morula , PCM Partially compacted morula , OR Odds ratios, CI Confdence intervals
